# Genetic Alterations and Therapeutic Targeting of Philadelphia-Like Acute Lymphoblastic Leukemia

**DOI:** 10.3390/genes12050687

**Published:** 2021-05-01

**Authors:** Ilaria Iacobucci, Kathryn G. Roberts

**Affiliations:** Department of Pathology, St. Jude Children’s Research Hospital, Memphis, TN 38105, USA; kathryn.roberts@stjude.org

**Keywords:** acute lymphoblastic leukemia (ALL), BCR-ABL1–like ALL, Ph-like ALL, cryptic rearrangements, kinase signaling, targeted therapy, immunotherapy

## Abstract

Philadelphia-like (Ph-like) acute lymphoblastic leukemia (ALL) is a subgroup of B-cell precursor ALL which by gene expression analysis clusters with Philadelphia-positive ALL although lacking the pathognomonic BCR-ABL1 oncoprotein. Its prevalence increases with age and similar to *BCR-ABL1*-positive ALL, Ph-like ALL is characterized by *IKZF1* or other B-lymphoid transcription factor gene deletions and by poor outcome to conventional therapeutic approaches. Genetic alterations are highly heterogenous across patients and include gene fusions, sequence mutations, DNA copy number changes and cryptic rearrangements. These lesions drive constitutively active cytokine receptor and kinase signaling pathways which deregulate ABL1 or JAK signaling and more rarely other kinase-driven pathways. The presence of activated kinase alterations and cytokine receptors has led to the incorporation of targeted therapy to the chemotherapy backbone which has improved treatment outcome for this high-risk subtype. More recently, retrospective studies have shown the efficacy of immunotherapies including both antibody drug-conjugates and chimeric antigen receptor T cell therapy and as they are not dependent on a specific genetic alteration, it is likely their use will increase in prospective clinical trials. This review summarizes the genomic landscape, clinical features, diagnostic assays, and novel therapeutic approaches for patients with Ph-like ALL.

## 1. Introduction

Acute lymphoblastic leukemia (ALL) comprises over twenty different subtypes defined by distinct constellations of somatic genetic alterations that converge on distinct gene expression profiles [1]. These include subtypes defined by recurring chromosomal abnormalities including aneuploidy, chromosomal rearrangements and/or known gene fusions; subtypes defined by cryptic rearrangements not identifiable by conventional approaches or by single point mutations; or novel subtypes that “phenocopy” established subtypes, with similar gene expression profile but different founding alteration [1,2,3,4]. One such subtype is Philadelphia chromosome-like (Ph-like) or BCR-ABL1-like ALL that is characterized by a gene expression signature similar to that of Philadelphia chromosome-positive ALL (Ph+ ALL), but lacking the pathognomonic BCR-ABL1 oncoprotein of Ph + ALL [5,6]. Similar to patients with Ph+ ALL, patients with Ph-like ALL often exhibit adverse clinical features and poor outcome [4,7,8] and frequently harbor alterations of *IKZF1* or other B-lymphoid transcription factor genes [6]. Multiple and heterogenous genetic alterations in kinases and cytokine receptors drive constitutively active kinase signaling which is amenable to targeted treatment with tyrosine kinase inhibitor (TKI) therapy [7,8]. Incidence of Ph-like ALL ranges from 10–15% in children [9,10] to 20% in older adults [11,12], with a peak (25–30%) in adolescent and young adult (AYA) population [4,13]. Due to the associated adverse prognosis and potential responsiveness of these patients to TKIs, the 2016 revision to the World Health Organization (WHO) classification of myeloid neoplasms and acute leukemia recognized Ph-like ALL as a provisional entity [14]. This review describes the current state of art in Ph-like ALL, highlighting the genomic landscape, clinical features, diagnostic assays, and therapeutic implications.

## 2. Clinical Features of Ph-Like ALL

### Prevalence and Outcome

The prevalence of Ph-like ALL increases with age. In children it accounts for up to 10% in National Cancer Institute (NCI) standard-risk B-ALL (SR; children age 1 to less than 10 years and white blood cell (WBC) count ≤ 50,000/μL) and up to 15% in NCI high-risk B-ALL (HR; age 10 to 16 years and/or WBC count ≥ 50,000/μL), and confers poor outcome [4,11,12]. In NCI HR childhood protocols from the Children’s Oncology Group (COG), the 5-year event-free survival was 58% compared to 84% for non-Ph-like ALL [4,15]. The prevalence of Ph-like ALL increases to approximately 20% in adolescents aged 16–20 years, reaches its peak (25–30%) in young adults aged 21–39 years and stabilizes at 20–24% in patients aged 40 to 86 years in the Unites States [4,10,11,12,13]. However, the prevalence of Ph-like in adults older than 40 years of age may vary according to the screened risk groups, the race and ethnicity of the patients studied, and the methodologies used to define Ph-like. For example, among adult patients with newly diagnosed B-ALL and gene expression profiling who received frontline chemotherapy at MD Anderson Cancer Center, 33.1% were Ph-like, of which 68% were of Hispanic ethnicity [12]. These patients had significantly worse overall survival and event-free survival compared with non-Ph-like B-ALL with a 5-year survival of 23% (vs. 59% for non Ph-like B-ALL) [12]. In European cohorts the prevalence of Ph-like ALL is lower with 17% of patients aged 16 to 71 years (of those 29% were patients aged 16–20 years; 43% were patients aged 21–39 and 29% patients aged 40–71 years) in a study from the Dutch-Belgian Hemato-Oncology Cooperative Group (HOVON) [16]. Ph-like patients had a high rate of non-response to first-line treatment and a high relapse rate compared to other B-ALL subtypes. However, Ph-like patients who underwent allogeneic stem cell transplant (ASCT) had a lower cumulative incidence of relapse at 5 years compared with patients without a donor. Only one relapse was seen among five patients who underwent ASCT [16]. In the report from the German Multicenter Study Group for Adult ALL trials (GMALL), GMALL 06/99 e 07/03, prevalence of Ph-like ALL was ~20% in adolescents and young adults but it decreased to under 10% in adults aged 40–84 years. This study reported 100% complete remissions even for Ph-like ALL, although with a short duration, lower number of molecular remissions and a higher rate of early relapses, confirming the negative prognostic impact on survival of the Ph-like ALL subtype in intensively treated older adults [17]. A similar prevalence (~20%) and poor clinical outcome were observed in patients enrolled in different GIMEMA (Gruppo Italiano Malattie EMatologiche dell’Adulto) clinical trials [18], where patients with Ph-like ALL had a lower rate of complete remissions and a worse event free survival compared with non Ph-like ALL cases [18,19].

Overall, regardless of age and study cohort almost all studies on patients with Ph-like ALL report adverse clinical features and inferior outcomes. Ph-like ALL cases tend to present with high WBC counts, are more commonly male, with a male to-female ratio of ∼2:1, and are associated with elevated minimal residual disease (MRD) levels, a high rate of treatment failure, and poor overall survival compared to non Ph-like ALL patients [4,10,11,12,13,15,16,17,20]. The only exception so far is from St. Jude Total XV, where the adverse prognosis of pediatric Ph-like ALL was improved by effective risk-directed therapy based primarily on MRD levels during and at the end of remission induction therapy [9]. Notably, in contrast to other studies that selectively examined high-risk B-ALL and did not apply MRD measurement for risk-directed therapy, this study included all patients with newly diagnosed ALL and used MRD level to direct intensity of therapy. Moreover, this study had a lower frequency of *CRLF2* (27.5%) and *IKZF1* (27%) alterations in Ph-like ALL than in prior studies from the COG [3,6,17]. The improved outcome from St. Jude Total XV contrasts with the Australian and New Zealand Children’s Haematology/Oncology Group (ANZCHOG) ALL8 clinical trial [21], where despite a risk-adjusted treatment approach, a high rate of disease recurrence was reported among children who were retrospectively diagnosed with Ph-like ALL.

## 3. Genomic Features of Ph-Like ALL

**Figure 1:**
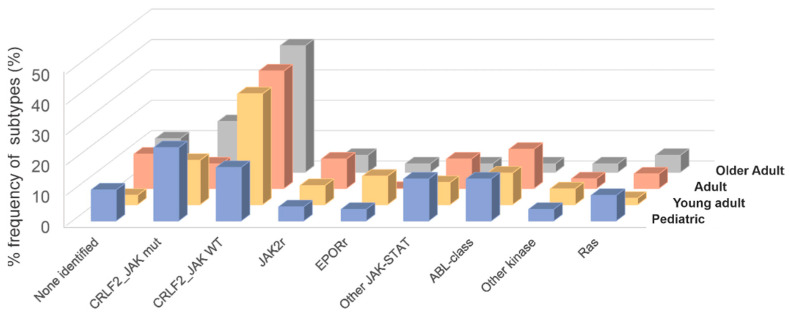
Frequency of Ph-like ALL subtypes according to each age group. Combined prevalence of Ph-like ALL subtypes in childhood (age 1 to 15 years), adolescent (age 16 to 20 years) and young adults (age 21 to 39 years), adults (age 40 to 59 years) and older adults (age > 60 years). Genomic subtypes include *CRFL2*-rearranged JAK mutant (mut), *CRFL2*-rearranged JAK wild-type (WT), *JAK2* rearrangements (JAK2r), *EPOR* rearrangements (EPORr), Other JAK-STAT alterations, ABL1-class fusions (*ABL1, ABL2, CSF1R, LYN, PDGFRA* and *PDGFRB*), Ras mutations (*KRAS, NRAS, NF1, PTPN11, BRAF* and *CBL*), all other kinase lesions (*FLT3, FGFR1, NTRK3*) and unknown alterations.

**Figure 2:**
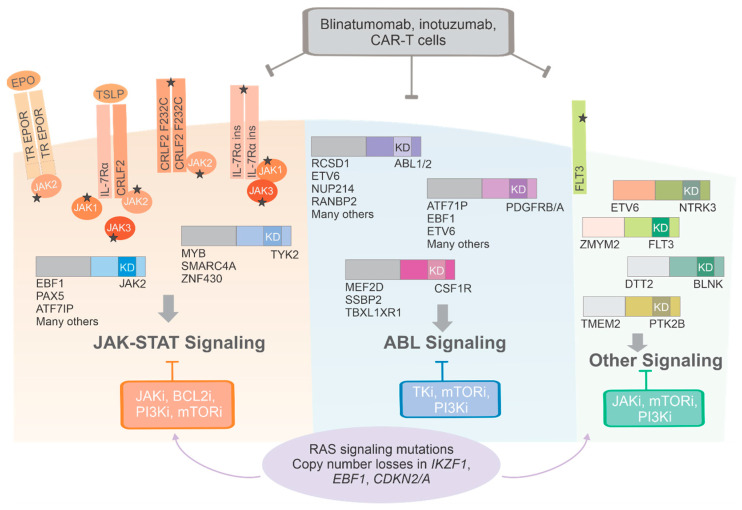
Schematic representation of main genomic alterations in Ph-like ALL. Constellations of sequence mutations, cryptic rearrangements, chimeric gene fusions and copy number changes drive constitutive cytokine receptor and kinase signaling which is amenable to inhibition by a variety of tyrosine kinase inhibitors. The majority of alterations converge on two pathways that activate JAK- family member signaling or ABL-signaling. Immunotherapeutic agents are not dependent on specific genetic alterations and represent promising approaches that warrant further investigation in larger trials. Abbreviations: TR: truncated; JAKi, JAK inhibitors; BCL2i, BCL2 inhibitors; PI3Ki, phosphoinositide 3-kinase inhibitor; mTORi, mTOR inhibitors; TKi, tyrosine kinase inhibitors. The star sign indicates the occurrence of a mutation.

A wide spectrum of genetic alterations (>60), including translocations, cryptic rearrangements, sequence mutations and copy number changes have been described in Ph-like ALL, with slight differences in prevalence across age (Figure 1). These alterations drive constitutively active kinase or cytokine receptor signaling, many of which have been shown to be druggable with a variety of kinase inhibitors. The most commonly mutated pathways are the ABL and JAK-STAT pathways with multiple rearrangements and lesions that converge on downstream ABL/JAK-STAT signaling. Founder alterations may be grouped into three types: (i) JAK/STAT alterations including mutations activating cytokine receptors (e.g., *CRLF2* and *IL7R*); gene fusions hijacking cytokine receptor expression (e.g., *IGH-CRLF2* and *P2RY8–CRLF2*) [22,23,24,25]; gene fusions and/or mutations activating kinases (e.g., *JAK1, JAK2, JAK3, TYK2*); and rearrangements hijacking and truncating cytokine receptor expression (e.g., cryptic *EPOR* rearrangements) [24]; (ii) fusions involving ABL-class genes (*ABL1*, *ABL2*, *CSF1R*, *LYN*, *PDGFRA*, *PDGFRB*); (iii) less common fusions (*FLT3, FGFR1, NTRK3, PTK2B*) [7] whose number is growing with increasing sequencing studies of different cohorts (Figure 2).

### 3.1. JAK/STAT Alterations

Approximately 50% of patients with Ph-like ALL harbor rearrangements of the cytokine receptor- like factor 2 (*CRLF2*) gene, located on the pseudoautosomal region 1 (PAR1) of chromosomes Xp22 and Yp11 [4,12,15,24]. In normal conditions CRLF2 dimerizes with the α- subunit of interleukin- 7 receptor (IL7RA) to form a heterodimeric thymic stromal lymphopoietin receptor (TSLPR) which actives downstream JAK2/STAT5 and thePI3K/AKT/mTOR pathways [26,27,28] and is implicated in early B-cell development [29]. *CRLF2* deregulation results from three main mechanisms: (1) a cryptic rearrangement that juxtaposes *CRLF2* to the immunoglobulin heavy chain locus (IGH); (2) a focal deletion in the pseudoautosomal region of the sex chromosomes resulting in P2Y receptor family member 8 (*P2RY8*)-*CRLF2* fusion that positions *CRLF2* under the control of the *P2RY8* promoter; (3) and less frequently by an activating CRLF2 point mutation, F232C [13,17,22,23,25,30,31,32,33]. Rearrangements of *CRLF2* account for 24% of pediatric patients with NCI SR Ph-like ALL [9], 55% of children with HR disease [10] and 50% to 60% of adolescent and adult patients with Ph-like ALL cases [4,11,12,13,17,18]. *P2RY8-CRLF2* fusions occur more commonly in younger children and in patients with Down syndrome (DS) ALL [22,25], while *IGH-CRLF2* fusions are detected more frequently in older patients and patients of Hispanic ethnicity [34]. In a genome-wide association study of *CRLF2*-rearranged ALL, the inherited *GATA3* variant rs3824662 was associated with *CRLF2* rearrangement, *JAK* mutation, *IKZF1* deletion, variation in *GATA3* expression and increased risk of relapse [35]. This variant is markedly more common in patients of Hispanic ethnicity (~40%) or Native American (~50%) genetic ancestry, while is it detected in only 14% of Europeans [35,36]. The point mutation changing phenylalanine 232 to cysteine in CRLF2 has been identified in 9% of DS-ALL patients [25] and 21% of adult B-ALL patients [23]. In in vitro assays, the expression of CRLF2 F232C in the absence of co-expression of mutant JAK2 promotes JAK2 signaling activation and cell transformation [23,25,37]. *CRLF2* rearrangement and overexpression is associated with worse outcome compared to cases with lack of *CRLF2* alterations [15,34,38,39]. However, the poor prognostic impact of *CRLF2* overexpression is overcome by *BCR-ABL1*–like signature and *IKZF1* deletion in the Dutch Childhood Oncology Group trials and German Cooperative ALL trials [37]. In about half of *CRLF2*-rearranged pediatric Ph-like ALL cases, concomitant *JAK1* and *JAK2* (most commonly in the pseudokinase domain at R683) mutations occur. In adults, the frequency of JAK mutations in patients with *CRLF2* rearrangement is lower, with a ratio of 1:4 with *JAK* wild type [4,12,15,23]. In *JAK1* the most common mutation is represented by V658F which is the homolog of JAK2 V617F, hotspot in myeloproliferative neoplasms. Other alterations leading to JAK/STAT activation target *IL7RA*, *SH2B3*, *IL2RB*, and *TYK2* genes. Collectively these alterations are approximately two-fold higher in children (14%) compared to adolescents (5.0%), and adults (7.3%) [4,12,15]. *IL7RA* mutations occur in exon 6 and are mainly in-frame insertion/deletions in the juxtamembrane-transmembrane domain or, rarely, a serine-to-cysteine substitution at amino acid 185 in the extracellular domain [38]. Independent of *CRLF2* rearrangements, JAK-STAT signaling activation can result from *JAK2* (~7%) or erythropoietin receptor (*EPOR,* 5%) -rearrangements.

Over 20 different *JAK2* gene fusion partners have been reported (most commonly *EBF1*, *ETV6*, *PAX5*, and *BCR*), making *JAK2* the most promiscuous gene in Ph-like ALL. All fusions preserve the JAK2 kinase domain and result in STAT5 activation and growth factor independence, making cells expressing these fusions amenable to JAK2 inhibitors.

Common *EPOR* rearrangements involve juxtaposition or less frequently translocation of the *EPOR* gene in proximity of a strong enhancer, such as that of the immunoglobulin heavy (*IGH*) or kappa (*IGK*) loci, that drives its expression. Less frequent rearrangements involve insertion of *EPOR* into the upstream region of *LAIR1* or the *THADA* loci [4,39]. All these rearrangements clip off the C-terminal cytoplasmic tail, thus preserving the proximal tyrosine requested for activation and removing almost all tyrosine sites required for shutting off the receptor signaling and down-regulate and internalize the receptor. This leads to transformation in in vivo models and sensitivity to a variety of different JAK2 inhibitors in in vitro and in vivo models. While *IGH-EPOR* fusion due the translocation t(14;19)(q32;p13) can be detected by fluorescence in situ hybridization (FISH) [40], the other *EPOR* rearrangements are cryptic and challenging to detect without using next-generation sequencing (NGS) technologies. The prevalence of *EPOR* rearrangements has a peak in young adults (9%) compared to children and adolescents (5% and 3%, respectively). They are rarely detected in adults (1%) [13,39]. *JAK2* and *EPOR* rearrangements are associated with the poorest outcome compared with the other molecular Ph-like subtypes [12,13].

### 3.2. Fusions Involving ABL-Class Genes

The ABL-class gene fusions include rearrangements of ABL proto-oncogene 1 (*ABL1;* e.g., to *RCSD1, NUP214, LSM14A, ETV6, RANBP2, CENPC, FOXP1, SFPQ, SNX1, SNX2, SPTNA1, ZMIZ1, NUP153*), ABL proto-oncogene 2 (*ABL2;* e.g., to *RCSD2, PAG1, ZC3HAV1*), colony-stimulating factor 1 receptor (*CSF1R;* e.g., to *SSBP2*, *MEF2D, TBL1XR1*), platelet-derived growth factor receptor beta (*PDGFRB;* e.g., to *EBF1, ETV6, ATF7IP, SNX29, SSBP2, TNIP1, ZEB2, ZMYND8, NUMA1*) and *LYN (GATAD2A-LYN, NCOR1-LYN* [41]), with multiple partner genes, with *ABL1* and *PDGFRB* being the most common. The prevalence of these rearrangements is 17% in children, 9% in adolescents, 10% young adults and 9% older adults [4,12,14,15]. Patients with ABL-class fusions respond poorly to chemotherapy regimens, and the *EBF1-PDGFRB* fusion in particular is associated with induction failure [42,43,44]. All fusions preserve the tyrosine kinase of the ABL-class gene and promote constitutive kinase signaling that confers the ability to survive and grow independently of cytokine in vitro [45]. Imatinib, the dual ABL1/SRC inhibitor dasatinib or other TKIs inhibit the downstream signaling induced by each of these chimeric fusion proteins [4,46,47] and are currently used in clinical trials. The best and first example is provided by the inhibition of *EBF1-PDGFRB* fusion by imatinib [44,46,47,48,49]. The emergence of kinase domain point mutations may represent a potential mechanism of relapse in *EBF1-PDGFRB* or other kinase driven-subtypes in Ph-like ALL. Recently, the T681I gatekeeper mutation has been demonstrated to be the most common resistant mutation in *EBF1-PDGFRB* Ph-like ALL to both imatinib and dasatinib in in vitro screens and it was associated with a trend towards increased risk of relapse in patients harboring T681I subclones at diagnosis compared to T681I-negative patients [50].

### 3.3. Other Kinase Fusions and Genetic Aberrations

Around 5% of Ph-like ALL cases harbor gene fusions or mutations involving *NTRK3, BLNK, DGKH, PTK2B, FLT3, FGFR1, TYK2* and *SH2B3*. Among those, one percent of cases harbor the fusion between *ETV6* and *NTRK3* encoding a member of the tropomyosin receptor tyrosine kinase (TRK) family [51]. This fusion is not unique of Ph-like ALL since it has been identified in a range of hematological malignancies, such as acute myeloid leukemia [52], infantile sarcoma [53,54] and solid tumors [55,56,57,58]. In preclinical models, ETV6-NTRK3 has been shown to promote the development of an aggressive B-ALL and to be exquisitely sensitive to the TRK inhibitors larotrectinib (LOXO-101) or PLX7486 (Plexxikon) in both patient derived xenograft models and in B-ALL patients with ETV6-NTRK3 [55,59,60]. Recently, a clinical response to larotrectinib has been reported in an adult Ph-like ALL with cryptic ETV6-NTRK3 rearrangement and NRASGly12Asp mutation. The patient failed to respond to multiagent chemotherapy and relapse after investigational CD19-directed chimeric antigen receptor T-cell therapy with a clone positive for ETV6-NTRK3 but not anymore for the NRASGly12Asp mutation. The relapsed leukemia progressed with further chemo- and immunotherapy but showed substantial leukemic cytoreduction using the TRK inhibitor larotrectinib [61]. Fusions of the B Cell Linker Protein (*BLNK*) or *SLP65* gene to *DNTT* (also known as *TDT*) have been also described [13,62]. BLNK encodes a cytoplasmic adapter protein important for B-cell development and function by activating BCR downstream signaling [63], while *DNTT* encodes a encodes a template-independent DNA polymerase that catalyzes the addition of deoxynucleotides and that is highly expressed in normal and malignant pre-B and pre-T lymphocytes during early differentiation [64].

In addition to gene fusions, RAS pathway activating mutations or deletions (*KRAS, NRAS, NF1, PTPN11*) and copy number aberrations in genes involved in B-cell development (*IKZF1, PAX5, EBF1,* and *ETV6*) and cell cycle regulators (*CDKN2A/B*, *TP53*, *BTG1*, and *RB1*) are recurrent. Deletions in *IKZF1* occur in around 27% of pediatric cases and in approximately 70% of high-risk pediatric patients with ALL [4]. As in *BCR-ABL* positive ALL [6,59], *IKZF1* deletions confer a poor prognostic outcome [20]. *IKZF1* deletions are significantly more common in patients carrying kinase or cytokine receptor rearrangement (*IGH-CRLF2*) than a sequence mutation [4,13], especially in Hispanic/Latino (H/L) children with B-ALL (29% in H/L compared to 15% of non-Hispanic Whites) where both *IGH-CRLF2* translocation and *IKZF1* deletion provide a strong biological rationale for the higher death-rate H/L experience due to B-ALL [60].

## 4. Diagnosis of Ph-Like ALL

**Figure 3:**
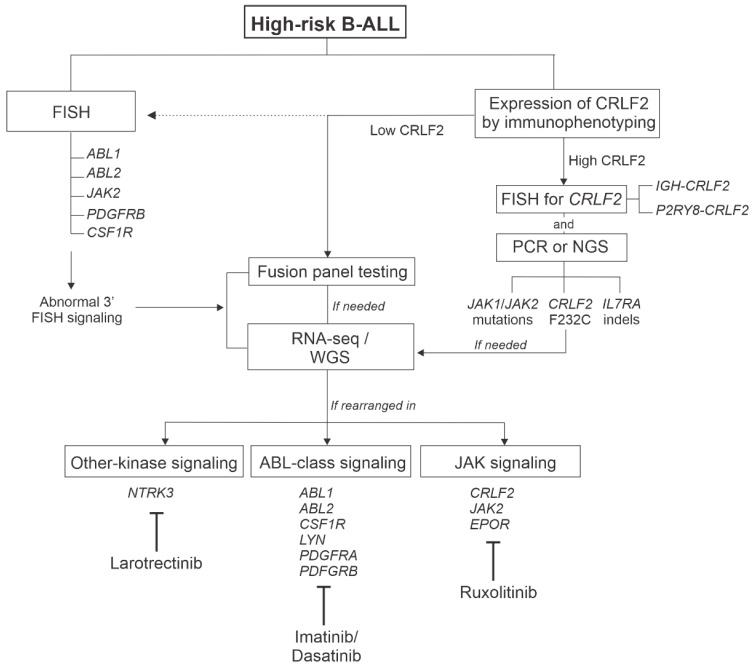
Flowchart of the current diagnostic approaches used to identify Ph-like ALL samples and summary of treatment options according to genetic lesions. Dotted line represents alternative approaches. Abbreviations: FISH, fluorescence in situ hybridization; PCR, polymerase chain reaction; NGS, next-generation sequencing; RNA-seq, whole transcriptome sequencing; WGS, whole genome sequencing.

As Ph-like ALL patients respond poorly to conventional chemotherapy, it has become increasingly important to diagnose these HR patients at presentation for improved therapeutic intervention. However, the heterogeneous genomic landscape and often cytogenetically cryptic alterations identified in Ph-like ALL creates a challenge for rapid and accurate diagnosis. Furthermore, there is no clear consensus on the definition of Ph-like ALL or the diagnostic methodologies used to identify these patients, and this has created confusion within the field [65]. Comprehensive clinical NGS, including whole transcriptome sequencing (RNA-seq), is the best approach to identify Ph-like ALL patients with targetable kinase alterations. Until these methodologies become widely available in the clinical setting, a tiered screening approach using routine diagnostics, including flow cytometry and fluorescence in situ hybridization (FISH), is still effective for swift identification of Ph-like ALL (Figure 3), as discussed below.

The original definition of Ph-like ALL was based on microarray gene expression profiling that identified cases with a similar gene expression signature to Ph+ ALL but that lacked the *BCR-ABL1* fusion [5,6]. Subsequent studies identified the diverse array of additional kinase-activating alterations that comprise this subtype, and that are potentially targetable with TKIs [4,12,15]. Whilst gene expression profiling can be used as an initial screening tool to flag potential Ph-like ALL patients [18,66], as clinical studies have evolved over the last decade it has become increasingly clear that identification of the underlying therapeutically targetable rearrangement itself is more important than determining the Ph-like gene expression signature.

Conventional cytogenetic analysis and FISH studies are routinely performed in the diagnostic work-up of newly diagnosed ALL patients. Although karyotypic analysis can identify major chromosomal rearrangements such as the Philadelphia chromosome (t(9;22) resulting in *BCR-ABL1*), the majority of Ph-like ALL rearrangements are cytogenetically cryptic. However, cytogenetics and FISH can be used to rule out known molecular subgroups that are usually mutually exclusive from Ph-like ALL (e.g., aneuploidy, *ETV6-RUNX1*, *BCR-ABL1*, *KMT2A*-rearranged, *TCF3-PBX1*). In addition, clinical break-apart FISH probes have been designed for the major 3′ kinase genes rearranged in Ph-like ALL, including *ABL1*, *ABL2*, *CRLF2*, *JAK2* and *PDGFRB* (which can also detect rearrangement of *CSF1R*). These assays can be performed with rapid turn-around and usually within a few days after the initial ALL diagnosis. Although FISH does not specify the 5′ partner gene of the kinase fusion, an abnormal result can be used to flag cases for downstream molecular analysis, and importantly, to facilitate entry onto clinical trials and early therapeutic intervention. Flow cytometric immunophenotyping for increased surface expression of TSLPR (encoded by *CRLF2*) is a highly cost-effective and predictive assay for detecting rearrangement of *CRLF2* (both *IGH-CRLF2* and *P2RY8-CRLF2*) and *CRLF2* F232C missense mutation in primary ALL blasts [28], and is becoming routinely incorporated into diagnostic panels. A positive staining for TSLPR should be followed up with FISH to confirm the specific *CRLF2* rearrangement. In *CRLF2*-rearranged cases, DNA-based polymerase chain reaction (PCR) assays can be used to determine the presence of *JAK1*, *JAK2* and *IL7R* sequence mutations, if desired.

Flow cytometric staining for TSLPR and FISH for break-apart kinase genes are used primarily as a rapid screening tool to identify cases that are eligible for therapeutic intervention, and that require further molecular characterization to determine the specific kinase rearrangement. This can be achieved using capture-based targeted sequencing platforms or clinical NGS, both of which provide comprehensive information on the chimeric fusion gene present. The FoundationOne Heme panel is a targeted combined RNA- and DNA-based capture that detects common fusions and mutations in over 400 cancer-related genes, including those identified in Ph-like ALL [67]. Many institutional laboratories have adopted the Archer FusionPlex Heme panel as it has become more widely available, is cost effective, and does not require comprehensive bioinformatic analyses. This is an anchored multiplex PCR-based assay that targets 87 genes identified in hematological malignancies, and importantly, can identify both known and novel 5′ partner genes [68]. Clinical RNA-seq is considered the gold standard to identify most kinase rearrangements in Ph-like ALL. This approach is also considered to be the slowest, most expensive and most intensive with regard to bioinformatic analyses. However, clinical NGS will likely replace targeted platforms as it becomes faster and more widely implemented. For example, the current frontline ALL treatment protocol at St Jude Children’s Research Hospital, Total XVII (NCT03117751), incorporates established morphologic, immunophenotyping and molecular genetic assays with comprehensive NGS diagnostics of both tumor and germline samples (whole-genome, whole exome and RNA-seq) performed in a CAP/CLIA-accredited laboratory as the clinical standard of care for all consented patients. Rapid RNA-seq reports rearrangements by day 15 of remission induction. Tumor and germline sequence information are also interrogated for copy-number variations, structural variations, single-nucleotide variants and insertion/deletions, which are reported by days 28–42 of remission induction [69].

## 5. Treatment Strategies for Ph-like ALL

### 5.1. Tyrosine Kinase Inhibitors

Whilst improvements in risk stratification and chemotherapy intensification has traditionally been an effective strategy for improving the outcomes of ALL, it is likely that this approach is reaching a point where the risk of toxicities outweighs the benefit of improved survival. For example, whilst the outcomes of adolescents and young adults (AYAs) were significantly improved with the pediatric-inspired CALGB 10403 regimen, the presence of Ph-like ALL remained an independent poor prognostic factor [70]. Therefore, new therapeutic strategies are required for these HR patients. The presence of activated kinase alterations and cytokine receptors in the majority of patients with Ph-like ALL has led to significant interest in incorporating TKIs in the clinical management of this subgroup, fueled by the success of adding TKIs to chemotherapy in patients with Ph+ ALL [71,72,73,74,75]. The two common subgroups of rearrangements in Ph-like ALL are ABL-class fusions (including *ABL1*, *ABL2*, *CSF1R*, *LYN*, *PDGFRA* and *PDGFRB*) and those activating JAK-STAT signaling (*CRLF2*, *JAK2* and *EPOR*) (Figure 3). For *ABL*-class fusions, preclinical studies have demonstrated encouraging anti-leukemic activity of TKIs in vitro and in patient-derived xenograft models [4,45]. Furthermore, anecdotal reports and case series have shown promising activity of either imatinib or dasatinib in patients with ABL-class fusions, either in the context of slow responders, as salvage for relapsed/refractory disease or as preemptive maintenance therapy post allogenic hematopoietic cell transplant in patients with persistent MRD [44,50,51,76,77]. Among 21 slow responders with an ABL-class fusion treated on UKALL2011, 13 were identified prospectively and treated with imatinib. The four-year relapse/refractory rate for the TKI group was 0% compared to 62.5% for the control group, with event-free survival rates of 83.9% vs. 37.5%, respectively [78]. Superior outcome was also observed in ABL-class fusion patients treated with TKI (imatinib or dasatinib) compared to chemotherapy alone on the AIEOP-BFM or FRALLE/GRAALL protocols [79,80]. Several large nonrandomized trials are currently ongoing to determine whether up-front addition of dasatinib to chemotherapy in children and AYAs decreases relapse risk and improves overall survival in Ph-like ABL-class fusion patients, including COG AALL1131 (NCT01406756) and SJCRH Total XVII (NCT03117751). Furthermore, the international AALL1631/EsPhALL2017 trial testing imatinib was amended to include patients with ABL-class Ph-like ALL (NCT03007147), and another international phase 3 trial for children with ABL-class Ph-like and Ph+ ALL is in development. A phase 1/2 international trial, AALL1922 (NCT04501614), will test the safety and efficacy of the third generation ABL1-inhibitor, ponatinib, in children and AYAs (1–21 years) with relapsed/refractory Ph+ ALL and Ph-like ABL-class ALL.

Preclinical studies have shown variable activity of JAK inhibitors (most commonly, ruxolitinib) in JAK-STAT activating ALL [39,81], and more recent studies demonstrate the need to inhibit multiple pathways in *CRLF2*-rearranged ALL, including PI3K and “BCR-like” signaling, to facilitate complete eradication of leukemic blasts [82,83]. Whilst several case reports have shown clinical activity for ruxolitinib in combination with chemotherapy in *JAK2*-rearranged ALL [84], convincing evidence of single agent activity in this setting is largely missing. The COG is running the largest trial testing the safety and efficacy of ruxolitinib in children and AYAs with newly diagnosed Ph-like ALL and *CRLF2*, *JAK2* or *EPOR* rearrangements via the single-arm phase 2 trial AALL1521 (NCT02723994). Phase 1 of this trial demonstrated the safety of combining ruxolitinib with chemotherapy [76], whilst phase 2 is currently assessing the efficacy of ruxolitinib at a dose of 50 mg/m^2^ administered in cycles of 14 days-on followed by 14 days-off. The University of Chicago is also recruiting for a phase 1 trial studying ruxolitinib in combination with the CALGB 10403 chemotherapy regimen (NCT00558519) [70] specifically in AYA patients (18–39 years of age) with JAK-STAT activating alterations.

### 5.2. Novel Therapies

Whilst TKI plus chemotherapy trials of Ph-like ALL are the logical first step to improve therapeutic outcome in these patients, the next generation of Ph-like ALL trials will likely incorporate novel immunotherapies, which have been remarkably effective in the treatment of relapsed/refractory B-ALL in children and adults irrespective of high-risk genetics or response to prior chemotherapies. This new treatment paradigm of combining targeted and immune therapies is supported by the promising results of the D-ALBA trial assessing blinatumomab plus dasatinib in newly diagnosed adults with Ph+ ALL [77]. Although published studies of immune therapies have not directly examined their efficacy in Ph-like ALL, retrospective studies provide some insight into their activity that warrant further investigation in larger trials. In a case series of 42 adults with relapsed/refractory B-ALL treated at City of Hope with the CD3/CD19 bispecific antibody, blinatumomab, 23 were found to have Ph-like ALL with an encouraging CR/CRi rate of 75% for *CRLF2*-rearranged and 57% for non-*CRLF2* patients compared to 33% for patients with non Ph-like ALL [85]. In another small series of patients treated at MDACC with the CD22 antibody drug-conjugate, inotuzumab, the response rate was comparable for Ph-like and non-Ph-like patients, suggesting this agent might at least abrogate the high rates of treatment failure observed with chemotherapy in Ph-like ALL [86]. Ongoing randomized frontline trials investigating the benefit of adding either blinatumomab (E1910 study; NCT02003222) or inotuzumab (A041501 Alliance study; NCT03150693) to chemotherapy regimens in adults with newly diagnosed Ph-negative ALL will provide valuable insights into the use of these novel therapies in Ph-like ALL. Chimeric antigen receptor (CAR) T cell therapy targeting CD19 has also shown remarkable results in relapsed/refractory B-ALL in children and adults, and whilst no studies to date have formally assessed outcomes in Ph-like ALL, it is likely that a significant number of patients enrolled on these trials were Ph-like, from which the response rate can be determined. Of the 4 children with relapsed/refractory Ph-like ALL treated with CD19 Car T cell therapy at Seattle Children’s Hospital, all responded and achieved MRD-negative CR. Notably, one patient received dasatinib post-CAR T therapy and two patients received HCT, indicating a combined therapeutic approach is required for the effective elimination of leukemic blasts in Ph-like ALL [87]. Analysis of larger CAR T cell trials will inform the benefit of this therapy in the treatment of Ph-like ALL.

## 6. Conclusions

The advent of integrated transcriptome and genomic studies have shed light on the genetic landscape of Ph-like ALL contributing to the identification of a wide range of targetable lesions previously not recognizable by standard diagnostics. Due to the complex heterogeneity of genetic alterations, diagnosis is challenging by conventional cytogenetic and/or molecular approaches and requires the use of comprehensive assays for detection of gene fusions, cryptic rearrangements, sequence mutations and copy number changes. Several commercial assays, mostly capture-based, have been developed and they are widely used in many institutional laboratories. The identification of specific genetic lesions is crucial for guiding targeted therapeutic intervention as the incorporation of tyrosine kinase inhibitors in the clinical management of these patients have led to significant improved outcomes. Lastly, the use of immunotherapeutic agents, such as blinatumomab, inotuzumab, and CAR-T cells, represents a promising alternative approach which is irrespective of a specific genetic alteration or response to prior chemotherapies.

## Data Availability

Not applicable.
